# Identification of a New Variant of the MBTPS1 Gene of the Kondo-Fu Type of Spondyloepiphyseal Dysplasia (SEDKF) in a Saudi Patient

**DOI:** 10.1155/2022/5498109

**Published:** 2022-10-25

**Authors:** Maha Alotaibi, Ali Aldossari, Imran Khan, Leena Alotaibi

**Affiliations:** ^1^Department of Genetic, Children's Hospital, King Saud Medical City, Riyadh, Saudi Arabia; ^2^Department of Orthopedic, Children's Hospital, King Saud Medical City, Riyadh, Saudi Arabia; ^3^Collage of Medicine, King Saud Bin Medical Abdul-Aziz University for Health Sciences, Riyadh, Saudi Arabia

## Abstract

Spondyloepiphyseal dysplasia (SEDKF) is a rare skeletal dysplasia associated with kyphosis and low bone mineral density, significantly delayed growth, and skeletal deformities. Blood lysosomal enzyme levels have also been shown to be elevated with a delay in development. The first variant described was compound heterozygosity for mutations in the MBTPS1 gene: a 1-bp duplication and a missense mutation. In the current study, we examined a Saudi consanguineous family. Clinical features like spondyloepiphyseal dysplasia, indicative of characteristic skeletal abnormalities, and impaired cognitive abilities were observed. Our patient has dysmorphic facial features, short stature, and significant skeletal deformities. A homozygous missense MBTPS1 (c.2634C > A p. (Ser878Arg)) with unknown significance was discovered in the whole exome; pathogenic MBTPS1 variants cause the autosomal recessive Kondo-Fu type of spondyloepiphyseal dysplasia (SEDKF, OMIM®: 618392). The whole exome sequence, which described a homozygous missense variant of unknown clinical significance (VUS, class 3 variant) in the MBTPS1 gene, was heterozygous in both asymptomatic parents. We are mindful that changing the classification of a variant of unknown significance is challenging. Considering clinical phenotypes and radiological findings produced by the pathogenic mutation in the MBTPS1 gene, the identified c.2634C > A variant is supported and may be categorized as likely pathogenic based on clinical symptoms.

## 1. Background

A diverse range of heritable disorders known as skeletal dysplasia causes bone deformities and cartilage growth, which alters the skeleton's size and shape, and causes disorders in long bones and spine. Spondyloepiphyseal skeletal dysplasia is a rare skeletal dysplasia that belongs to the cellular protein transport abnormalities disorders, which are characterized by cellular protein transport abnormalities. As a result, improperly accumulated macromolecules lead to cellular stress and skeletal abnormalities. The membrane-bound transcription factor peptidase, site 1 gene (MBTPS1, OMIM*∗*603355) encodes the Site 1 protease (S1P), which works in tandem with the Site 2 protease (S2P) as a proteolytic activator of membrane-bound latent transcription factors in the Golgi [1].

However, S1P has a function that is unrelated to S2P. GlcNAc-1-phosphotransferase activation has been demonstrated (GPT) and it is required to introduce mannose-6-phosphate (M6P) modification of lysosomal enzymes into the Golgi apparatus, and as a result, introduce M6P modification of lysosomal enzymes into the Golgi apparatus [2] and facilitates their trafficking to lysosomes.

Kondo-Fu type spondyloepiphyseal dysplasia (SEDKF) is an MBTPS1-related skeletal dysplasia with mild to severe intellectual disability, growth retardation, dysmorphic facial features, bilateral cataracts, short stature, kyphosis, waddling gait, and other skeletal abnormalities such as the irregular dysplastic appearance of femoral epiphyses, gracile fibulae, and valgus. Because of their rarity, these disorders are frequently misdiagnosed and their processes of action are poorly understood. Despite the fact that just a few cases have been reported, a group of physicians and scientists in Oklahoma, US, reported the first patient with an MBTPS1 gene mutation in 2018. Spondyloepiphyseal dysplasia was present in this case (SED, a condition that primarily affects the development of bones in the spine and the ends of long bones). The disorder was termed SED, Kondo-Fu type, after Drs. Yuji Kondo and Jianxin Fu, who wrote the published report. Since then, seven cases with SEDFK have been documented. However, no new cases have been recorded in the Middle East or Saudi Arabia. There is no treatment for this disease; only supportive care is available and its clinical and prognosis are also still unclear. The clinical characteristics and molecular genetic test results of one Saudi girl, the first reported case in the Middle East, are presented.

## 2. Case Presentation

**Figure 1:**
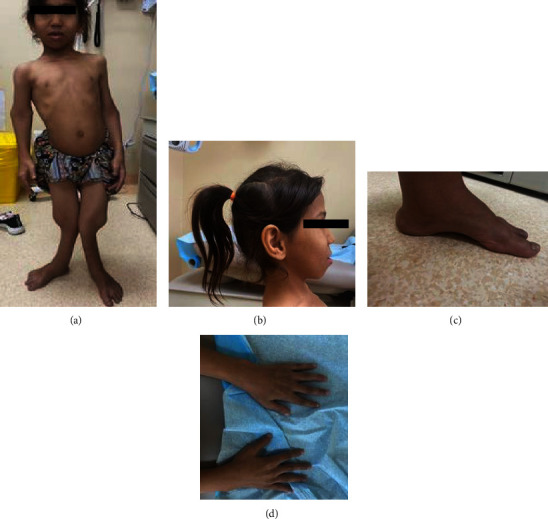
Clinical pictures. (A, B) Triangular face with prominent cheekbones, retromicrognathia, protruding external ears, short neck, pectus carinatum, protruding abdomen, shortening of limbs, and knock knee. (C, D) Pes cavus foot and brachydactyly.

**Figure 2:**
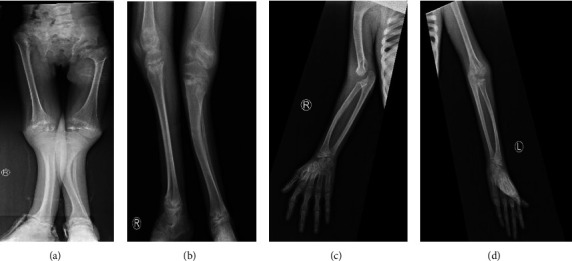
Radiographies. (A, B) Diffuse osteopenia and tubular bones are short. Metaphyseal sclerosis and irregularities more pronounced in and sclerotic femoral heads. Right hip dislocation with external the lower limbs. (C, D) Right humerus bowing deformity. Small, fragmented rotation of the right lower limb. Bilateral coxa varus. Left genu valgus. Bowing deformity of the left tibia. Metacarpals and metatarsal are short.

The patient was a 10-year-old girl at the time of her visit to the Department of Genetic Medicine, King Saud Medical City, Riyadh. She is the first child of the first healthy Saudi cousin. She was born at full term of pregnancy via spontaneous vaginal delivery. The family history was unremarkable, and the other four siblings were healthy, with a history of one abortion. She had early motor development delay (sat at 2 years and walked at 3 years), speech delay, and subnormal mentality. Skeletal changes with a waddling gait, difficulty rising after sitting down. During the clinical examination, she had good eye contact, obeyed simple commands, and could pronounce a few unclear words. She could walk with assistance. Stairs are difficult. She looks like a dysmorphic triangular face with a prominent check, a forehead, retromicrognathia, large ears, and skeletal deformities with mild pectus carinatum, short and broad hands with brachydactyly, kyphosis, knock knees, valgus tibial bowing, and pes cavus foot (Figures 1(a)–1(d)), and her height was 102 cm (2.9 SDs), weight was 16 kg, and head circumference was 48 cm below the third percentile. The radiological findings (Figures 2(a)–2(d)) coincided with the clinical findings. The radiological analysis revealed diffuse osteopenia. Tubular bones are short. Metaphyseal sclerosis and irregularities are more pronounced in the lower limbs. Right humerus bowing deformity; small, fragmented, and sclerotic femoral heads; right hip dislocation with external rotation of the right lower limb; bilateral coxa varus; and left genu valgus bowing deformity of the left tibia were identified. Metacarpals and metatarsals are short. The echocardiogram and abdominal ultrasound were unremarkable. She had an intellectual disability (IQ = 60) and lived alone. No corneal cataract, hearing deficit, or involvement of other systems was identified.

Investigations included complete blood count; coagulation profile; GAG (glycosaminoglycans) in the urine, urea, and electrolytes; thyroid function tests; parathyroid hormone; serum calcium; phosphorus; and alkaline phosphatase.

Magnesium, vitamin D, and liver enzymes were normal. Unremarkable Tandom MS was found, and urine organic acid analysis was performed. Lysosomal enzyme activities were analyzed and showed elevated enzyme activity levels for alpha-glucosidase, beta-glucuronidase, beta-mannosidase, alpha-mannosidase, and beta-hexosaminidase.

WeS was performed on the proband and her parents utilizing genomic DNA extract from blood. Genomic DNA is enzymatically fragmented, and target regions are enriched using DNA capture probes. These regions include approximately 41 Mb of the human coding exome (targeting >98% of the coding RefSeq from the human genome build GRCh37/hg19), as well as the mitochondrial genome. The generated library is sequenced on an Illumina platform to obtain at least 20x coverage depth for >98% of the targeted bases. An in-house bioinformatics pipeline, including read alignment to the GRCh37/hg19 genome assembly and revised Cambridge Reference Sequence (rCRS) of the Human Mitochondrial DNA (NC_012920), variant calling, annotation, and comprehensive variant filtering, is applied. All variants with a minor allele frequency (MAF) of less than 1% in the gnomAD database, as well as disease-causing variants reported in HGMD®, ClinVar, or CentoMD® are considered. The investigation for relevant variants is focused on coding exons and flanking ±10 intronic nucleotides of genes with clear gene-phenotype evidence (based on OMIM® information). All potential patterns for the mode of inheritance are considered. In addition, provided family history and clinical information are used to evaluate identified variants with respect to their pathogenicity and causality. Variants are categorized into five classes (pathogenic, likely pathogenic, VUS, likely benign, and benign) according to ACMG guidelines for classification of variants. All relevant variants related to the phenotype of the patient are reported. CENTOGENE has established stringent quality criteria and validation processes for variants detected by NGS. Variants with low quality and/or unclear zygosity are confirmed by orthogonal methods. Consequently, a specificity of >99.9% for all reported variants is warranted. Mitochondrial variants are reported to have heteroplasmy levels of 15% or higher.

The copy number variation (CNV) detection software has a sensitivity of more than 95% for all homozygous/hemizygous and mitochondrial deletions, as well as heterozygous deletions/duplications and homozygous/hemizygous duplications spanning at least three consecutive exons. For uniparental disomy (UPD) screening, a specific algorithm is used to assess the well-known clinically relevant chromosomal regions (6q24, 7, 11p15.5, 14q32, 15q11q13, 20q13, and 20).

**Figure 3:**
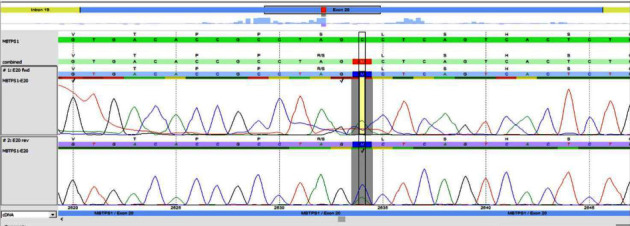
Molecular study of the genomic DNA of patient detected MBTPS1.

The patient's entire exome sequence was discovered to be a homozygous (c.2634C > A) p. (Ser878Arg) variant of the MBTPS1 gene (Figure 3). An amino acid change from Ser to Arg at position 878. It is classified as a variant of uncertain significance (class 3) according to the recommendations of CENTOGENE and ACMG, and further Sanger sequencing indicated that the parents carried a single heterozygous mutation each. The missense c.2634C > A variant in the MBTPS1 gene has not been previously reported in clinical cases and was classified as a Variant of Uncertain Significance (class 3) according to the CENTOGENE and ACMG. The Kondo-Fu type of spondyloepiphyseal dysplasia (SEDKF) is caused by a homozygous mutation in the MBTPS1 gene. The autosomal recessive Kondo-Fu type of spondyloepiphyseal dysplasia (SEDKF) is consistent with the genetic diagnosis of the autosomal recessive Kondo-Fu type of spondyloepiphyseal dysplasia (SEDKF). Variable clinical phenotypes include short stature, developmental delay, eye abnormalities, and skeletal dysplasia. We are aware of the paper published by Kondo et al. [3]. They report cases of MBTPS1 variants identified by compound heterozygosity for mutations in the MBTPS1 gene: a 1-bp duplication and a missense mutation. The gene variant mentioned here has never been described in the literature before.

## 3. Discussion

Skeletal dysplasia is a heterogeneous group of bone growth disorder, nonfatal, presented as short stature and skeletal deformities [4]. Clinically and genetically, these disorders vary widely. While some spondyloepiphyseal dysplasia, such as spondyloepiphyseal dysplasia congenita (SEDC), appear during pregnancy, others do so during childhood.

The Kondo-Fu form of spondyloepiphyseal dysplasia SEDKF is one of the rare disorders that come under the umbrella of skeletal dysplasia. One patient with SEDKF was included in our investigation. Short trunk and intellectual impairment are all manifestations of SEDKF. Dysmorphic facial features, kyphosis, pectus carinatum, and bone deformity are further SEDKF symptoms. During the clinical valuation of our patient, including body examination and radiological investigations, we did not manifest bilateral cataract and inguinal hernia.

Membrane-bound transcription factor site-1 protease (MBTPS1) is an enzyme encoded by the MBTPS1 gene in humans. S1P cleaves the transcriptional regulators of the sterol regulatory element-binding protein's endoplasmic reticulum loop (SREBP) [5]. Due to genetic abnormalities in key regulators of the secretory apparatus, MBTPS1-related disorders are a category of hereditary diseases defined by secretion deficits in chondrocytes in cartilage, resulting in skeletal dysplasia and eventual growth retardation. If too much collagen is not released from cells and eventually accumulates within the cells, the skeletal organs can malfunction.

Spondyloepiphyseal dysplasia of the Kondo-Fu type (SEDKF) is a rare autosomal recessive disorder caused by a mutation in the MBTPS1 gene that results in short stature and other phenotypes such as a triangular face, spondyloepiphyseal dysplasia, kyphosis, growth retardation, delayed motor milestones, and elevated lysosomal enzymes.

Here, the first Arab patient with an MBTPS1 mutation has been identified, and pathogenic variants in this gene have been linked to clinical features of the Kondo-Fu type of spondyloepiphyseal dysplasia (SEDKF). Indeed, the patient described by Kondo [3] with the different variant and our patient share many clinical characteristics. In both patients, growth is retarded, but it is more severe in our case. Both patients had the same facial phenotype (high forehead, prominent cheekbones, large ears, and skeletal deformities such as pectus carinatum, kyphosis, and tibial varus bowing). Increased lysosomal enzyme levels were seen in our patient, which was consistent with biochemical findings from the literature and compatible with S1P deficiency. The parents are both carriers of the c.2634C > A variation.

A few phenotypes associated with MBTPS1 have been reported. In an adult with episodic hyperCKemia and focal myoedema, a heterozygous missense mutation (de novo) in the transmembrane domain of S1P (p.Pro1003Ser) was described [6]. A phenotype substantially different from the case has been reported by Kondo et al. [3] and our proband. Another case of Kondo-Fu type spondyloepiphyseal dysplasia (SEDKF) was reported with unique findings in a 5-year-old Brazilian girl with epilepsy and craniosynostosis [7].

To the best of our knowledge, this is the first case of the Kondo-Fu type of spondyloepiphyseal dysplasia (SEDKF) involving the homozygous c.2634C > A p. (Ser878Arg) variant of unknown clinical significance (VUS, class 3 variant) in the MBTPS1 gene, OMIM #618392, on chromosome 16q23-q24. The WES result revealed the presence of a missense substitution. Considering the patient's phenotype and the medical symptomatology of the syndrome generated by pathogenic mutations in the MBTPS1 gene, the identified c.2634C > A variant may be reclassified as a class 4 (likely pathogenic) mutation.

Thus, advanced genetic testing has enabled the identification and characterization of several genes involved in various genetic syndromes at the pathophysiological level. Thus, the report presents a rare case of the Kondo-Fu type of spondyloepiphyseal dysplasia (SEDKF) based on advanced genetic diagnostics, besides clinical examinations and radiological findings, and helps in providing proper supportive management to the patient, prenatal diagnosis, genetic counselling, and future pregnancy decision-making.

## Data Availability

All the data generated or analyzed during the study are included in this article.

## Abbreviations

TD: Thanatophoric dysplasia
FGFR 3: Fibroblast growth factor receptor 3
NICU: Neonate intensive care unit
APGARS: Appearance, pulse, grimace, activity, and respiration score
G3P2: Gravidity 3 and parity 2 mother.
